# Vibration Analysis at Castello Ursino Picture Gallery (Sicily, Italy) for the Implementation of Self-Generating AlN-MEMS Sensors

**DOI:** 10.3390/s24175617

**Published:** 2024-08-29

**Authors:** Claudia Pirrotta, Anna M. Gueli, Sebastiano Imposa, Giuliano A. Salerno, Carlo Trigona

**Affiliations:** 1 DSBGA, Dipartimento di Scienze Biologiche, Geologiche e Ambientali, University of Catania, Corso Italia 57, 95129 Catania, Italy; claudia.pirrotta@unict.it; 2 DFA, Dipartimento di Fisica e Astronomia “Ettore Majorana”, University of Catania, Via Santa Sofia 64, 95123 Catania, Italy; 3 DIEEI, Dipartimento di Ingegneria Elettrica Elettronica e Informatica, University of Catania, Viale Andrea Doria 6, 95125 Catania, Italy; giuliano.salerno@phd.unict.it

**Keywords:** Castello Ursino Museum, cultural heritage, MEMS, modal parameters, vibration monitoring

## Abstract

This work explores the potential of self-powered MEMS devices for application in the preventive conservation of cultural heritage. The main objective is to evaluate the effectiveness of piezoelectric aluminum nitride MEMS (AlN-MEMS) for monitoring vibrations and to investigate its potential for harvesting energy from vibrations, including those induced by visitors. A preliminary laboratory comparison was conducted between AlN-MEMS and the commercial device Tromino^®^. The study was then extended to the Picture Gallery of Ursino Castle, where joint measurements with the two devices were carried out. The analysis focused on identifying natural frequencies and vibrational energy levels by key metrics, including spectral peaks and the Power Spectral Density (PSD). The results indicated that the response of the AlN-MEMS aligned well with the data collected by the commercial device, especially observing high vibrational energy around 100 Hz. Such results validate the potential of AlN-MEMS for effective vibration measurement and for converting kinetic energy into electrical power, thereby eliminating the need for external power sources. Additionally, the vibrational analysis highlighted specific locations, such as the measurement point Cu4, as exhibiting the highest vibrational energy levels. These points could be used for placing MEMS sensors to ensure efficient vibration monitoring and energy harvesting.

## 1. Introduction

Monitoring procedures for the preventive conservation of infrastructures and assets are principally based on vibration analysis [1,2]. The reason for this is twofold: on the one hand, it is because vibrations can be a threat to the integrity of structures and assets, and on the other hand, because, through their analysis, it is possible to non-invasively assess a range of modal parameters indicative of the dynamic behavior of the structure and of the associated kinetic energy levels. It should be noted that, despite vibrations not directly causing primary damage, they possess the potential to exacerbate pre-existing critical structural conditions [3]. Additionally, vibrations can significantly impact the well-being of building occupants, whether in occupational or domestic settings [4]. Given that vibrations are consistently present in the environment, originating from natural phenomena like wind and earthquakes, as well as escalating human activities such as construction, motorized transportation, and industrial machinery, their continuous monitoring has become of paramount importance.

In the frame of Cultural Heritage, environmental vibration monitoring is an essential practice in preventive conservation strategies, aiming to preserve cultural heritage in its current state, preventing the need for more radical and costly restoration interventions. Within a museum environment, preventive conservation ensures the management and preservation of artworks and supporting structures such as display cases, revetments and frames [5,6]. By performing continuous monitoring, it is possible to identify and mitigate harmful vibration sources before they cause significant damage. Additionally, vibration monitoring allows for the real-time assessment of structural conditions by identifying variations in dynamic parameters, which can indicate the presence of damage or structural degradation. The collected data can be used to plan targeted maintenance and restoration interventions, optimizing available resources and minimizing invasive procedures.

Vibration analysis is foundational to Structural Health Monitoring (SHM), particularly for evaluating the dynamic behavior of structures. Operational Modal Analysis (OMA) emerges as a prominent technique within this context, leveraging vibration analysis to estimate modal parameters—such as natural frequencies, damping ratios, mode shapes, and kinetic energy distribution—that allow for the assessment and monitoring of the integrity and health of structures [7,8,9,10,11].

Based on vibrations, OMA is an advanced and minimally invasive technique. This approach eliminates the need for the highly controlled excitation conditions typically required in traditional methods, which often involve invasive examination procedures. Such procedures are particularly unsuitable for heritage structures due to their fragile nature and the need to preserve their structural integrity.

Monitoring systems employ various sensors and techniques such as gauges, photogrammetry, laser interferometry, fiber optic Bragg strain sensors (FBG) [12] and Brillouin optical analysis (BOTDA) [13,14], vibroacoustic sensors [15], and fast image-based techniques [16]. Vibration-based monitoring methods widely employ accelerometers [17,18] that offer significant benefits due to their affordability, durability, and ease of installation. The conventional devices used to capture ambient vibrations have two main limitations, particularly when performing continuous monitoring in cultural heritage contexts. First, these devices are bulky and highly visible, disrupting the environment and landscape, especially in cultural heritage sites. Additionally, they require an operator during the acquisition phases, interfering with the regular activities of a cultural site, such as a museum. Second, they need a power supply, and if they rely on batteries, these need to be regularly replaced. In the direction of integration and miniaturization, recent advancements in sensor technology have led to the development of micro and nano-scale sensors, such as Micro Electromechanical Systems (MEMSs), that offer significant advantages in vibration monitoring, detecting even minor displacement or deformation [19,20]. These miniaturized devices can be discreetly integrated into the environment, overcoming the problem of their visibility. Specifically, this innovation involves implementing devices capable of operating as self-powered sensors without the need for external power sources or batteries, allowing them to continuously record data. These sensors are equipped with energy-harvesting capabilities that enable them to generate power from their surrounding environment [21]. They can harness energy from various sources such as wind [22,23], temperature differences [24], or sunlight [25]. Recent research has focused on capturing vibrations as promising energy reservoirs [26], leading to the development of compact vibrational energy collectors. This self-generating ability makes MEMSs valuable for remote and autonomous sensing applications, where long-term and maintenance-free operations are essential. Their compact size, low power consumption, high sensitivity and cost-effectiveness make them invaluable for the long-term monitoring and preservation of infrastructures and particularly valuable in heritage sites.

The aim of this work is to investigate the capacity of self-powered AlN-MEMSs, designed to operate with kinetic energy, to capture environmental vibrations at the specific installation site, for dual future applications. This is firstly to assess the suitability of MEMSs for vibration monitoring in the development of an advanced monitoring system, and secondly, to explore the potential to harvest energy from ambient vibrations, including those induced by visitors. With these aims, the performance of the AlN-MEMS was compared to that of a commercial seismometer named Tromino^®^, developed by MoHo s.r.l. (Marghera, Venice, Italy) [27]. The latter was designed for the dynamic characterization of soils and the monitoring of structural vibrations for a wide range of applications, including civil engineering, geotechnical studies, and seismic hazard assessment [28,29,30,31,32]. In addition, the Tromino^®^ device was used to identify areas of higher vibration for the strategic positioning of self-generating AlN-MEMS devices.

**Figure 1:**
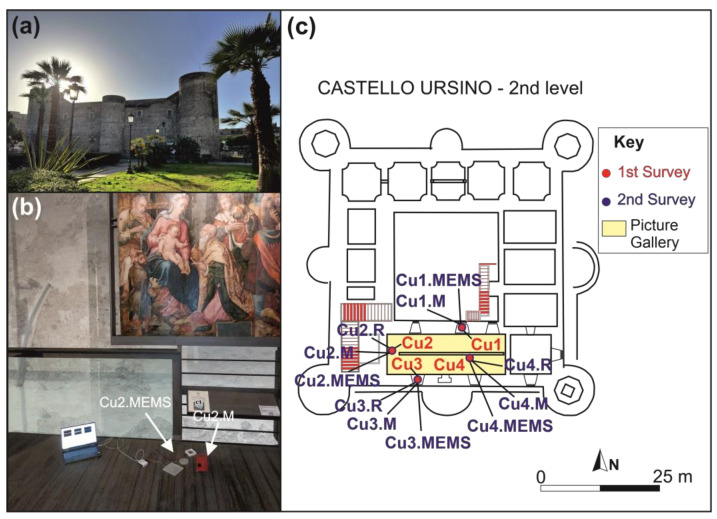
(**a**) Photo of Castello Ursino; (**b**) Simultaneous acquisition of measurements from the Tromino^®^ device (Cu2.M) and AlN-MEMS (Cu2.MEMS); (**c**) Map of the second floor of Castello Ursino indicating the positions of the vibration measurements. The red dots, from Cu1 to Cu4, refer to the acquisition points of the first survey. The blue dots indicate the acquisition points of the second survey: Cu2.R to Cu4.R refer to the data acquired exclusively by the Tromino^®^; Cu1.M to Cu4.M represent the data collected by the Tromino^®^ simultaneously with the AlN-MEMS; lastly, Cu1.MEMS to Cu4.MEMS indicate the data acquired by the AlN-MEMS simultaneously with the Tromino^®^.

Since the AlN-MEMS was to be employed in a monitoring system designed for cultural heritage preventive conservation, a museum environment was chosen as the test setting for this work. Here, vibrations are additionally induced by the presence of visitors and insiders. Specifically, this study focuses on analyzing the vibrations and kinetic energy distribution in the Picture Gallery of the Castello Ursino Museum, in Catania, Italy (Figure 1). This building is a medieval fortress, commissioned by Frederick II of Svevia in the late 13th century and holding historical significance as a former royal residence and parliamentary seat. Presently, the Castello Ursino hosts the Civic Museum of Catania with artworks of great importance. Besides its relevance from the point of view of cultural heritage, the Castello Ursino was chosen as the test site due to its architectonic complexity, making it a challenging case. The castle showcases great symmetry and distinctive architectural traits, marked by a quadrangular layout featuring sturdy towers (Figure 1a). Nonetheless, restoration efforts in 1932 altered its original architecture, resulting in a modernized version divided into four levels. Mezzanines were added to certain rooms during this renovation.

It is worth noting that a previous study also aimed to perform long-term vibration monitoring through an autonomous node composed of centimeter-scale piezoelectric elements [33]. This paper advances the state of the art by presenting a miniaturized transducer capable of measuring vibrations within the range of interest for preventive conservation (<100 Hz). Additionally, it could harvest energy from the vibrations in the monitored area, such as those induced by visitors, external kinetic motions, and noisy environments.

## 2. Vibration Analysis Methods

Vibration measurements were conducted using an OMA approach, which involves acquiring data under real-world operating conditions.

The acquired vibration data were processed to extract metrics in both the frequency-domain and the time-domain. Regarding both the data acquired by the Tromino^®^ and the AlN-MEMS, the Spectral Analysis (Fast Fourier Transform, FFT) and the Power Spectral Density (PSD) were computed to observe the natural frequencies and kinetic energy levels at specific points inside the Picture Gallery. This analysis serves as a compatibility verification phase between the AlN-MEMS sensor and the Tromino^®^. Demonstrating good compatibility would indicate the suitability of AlN-MEMS for vibration monitoring purposes. An additional aim of the analysis performed on the data acquired with the Tromino^®^ was to identify areas of significant vibrational energy that would be suitable for the self-powering mechanisms of the AlN-MEMS. For this reason, additional analyses were conducted on the data acquired by the Tromino^®^, such as the Acceleration Peaks Analysis and Root Mean Square (RMS), in the time-domain.

Data analyses were performed by creating apposite scripts in MATLAB; only the Spectral Analysis (FFT) of data acquired by the Tromino^®^ was performed by using the Grilla Software Rel. 9.8.5 (Moho, s.r.l., Marghera, Venice, Italy), specifically designed for this analysis.

Since the Tromino^®^ is a 3D velocimeter and the AlN-MEMS is a vertical accelerometer, only the vertical component of the data obtained from the Tromino^®^ was considered for the analysis. Additionally, given the instrumental differences between the Tromino^®^ and AlN-MEMS, specific treatments were applied to the signals acquired by each sensor before processing to optimize the comparison analysis. The pre-processing of the data acquired by the Tromino^®^ included demeaning and detrending to remove trends and subtract the mean value, respectively. This eliminated very low-frequency components and constant polarizations, improving the features and reliability of the signal processing. For the analysis of the data acquired by the AlN-MEMS, an 8th-order FIR low-pass digital filter with a cutoff frequency set at 200 Hz was applied. Additionally, Fast Fourier Transform and Power Spectral Density Analysis were applied to these data to investigate the performance in terms of the transduction capability of the proposed device [34,35].

### 2.1. Spectral Analysis (FFT)

In the context of structural monitoring, the acceleration spectrum, which shows the amplitude of acceleration at different frequencies of the signal [36], allows the dominant frequencies to be identified, indicating the predominant vibration modes and providing insights into the intensity of the dynamic forces acting on the structure.

Analyzing the frequency distribution detects the presence of resonant frequencies or vibration modes, contributing to a more comprehensive understanding of dynamic responses.

The vibration measurements acquired with Tromino^®^, as mentioned above, were processed with Grilla Software Rel. 9.8.5 (Moho, s.r.l.). Spectral analysis was conducted by applying the Fast Fourier Transform on 20-s windows, which were subsequently averaged. The resulting graph correlated the acceleration and frequency of the recordings, along with the respective standard deviation, enabling the verification of the acquired data’s variability.

### 2.2. Power Spectral Density Analysis

The PSD provides essential insights into how a signal’s energy is distributed across various frequency bands. It is extensively used in vibration analysis, signal processing and in examining the dynamic behavior of physical systems. Understanding the energy distribution through PSD is critical in these fields for analyzing and interpreting the frequency characteristics of signals. In this study, the PSD was analyzed in terms of the power of acceleration as a function of frequency, quantified in units of [(mm/s^2^)^2^/Hz].

To compute the PSD of vibration data, the Welch method was employed using MATLAB [35,37,38]. This method is well regarded for its capacity to enhance the frequency resolution, reduce variance, and address spectral leakage. The analysis involves the FFT of overlapping segments. The periodogram of each segment is calculated by squaring the magnitude of the FFT results (1).

(1) $$P_{x}\left(f\right)=\frac{1}{N}{\left|X\left(f\right)\right|}^{2}$$

In this formula, *P*_*x*_(*f*) is the periodogram, *X*(*f*) is the FFT of the segment, and *N* is the number of points in the FFT.

The output was graphically represented in a Frequency (Hz)–PSD [(mm/s^2^)^2^/Hz] plot, providing a comprehensive and insightful visualization of the inherent characteristics and spectral features of the seismic signals.

### 2.3. Acceleration Peak Analysis

The acquired vibration signals were reconstructed and graphically represented in a Time–Acceleration plot using MATLAB. The software enables the reconstruction of the signal by defining two crucial acquisition parameters: the sampling frequency and the total duration of the data recording in seconds. This graphical representation helps to identify Acceleration Peaks over time, characteristic patterns, or anomalies in the data.

The Acceleration Peak is a significant synthetic parameter, indicating the maximum positive or negative oscillation amplitude of the vibration, and is often associated with short-duration impacts. It is important to note that these maximum acceleration values may be influenced by the surrounding environment and can be linked to transient solicitations, such as the passage of people close to the sensor.

### 2.4. Root Mean Square Analysis

In vibration analysis, the Root Mean Square (RMS) (2) is a significant indicator of the amplitude of a vibrating wave. While the peak value represents the maximum displacement from the wave’s rest position, the RMS provides a measure of the effective amplitude, considering the acceleration distribution over time. Therefore, the RMS provides a more comprehensive assessment of the amplitude of a vibrating signal compared to the simple peak value.

The formula for calculating the RMS value is given by:

(2) $$\mathrm{RMS}=\sqrt{\frac{1}{T}\int_{0}^{T}{\left[x\left(t\right)\right]}^{2}\;\mathrm{dt}},$$

where *x*(*t*) represents the signal variation over time, and *T* is the duration of the signal.

In the case of digital signals, the RMS considers the sum of the squares of the signal samples, calculates the average, and then extracts the square root of the result.

(3) $$\mathrm{RMS}=\sqrt{\frac{1}{N}\sum_{i=1}^{N}{\left|x_{i}\right|}^{2}}$$

where x_i_ is the value of the *i*-th sample of the signal.

This process takes into account both impulses and continuous variations in the signal, providing an RMS value representative of the overall amplitude.

The RMS values were visualized on the Time–Acceleration graph as positive and negative horizontal lines. Specifically, these lines help in understanding the magnitude of and variation in the acceleration signal over time.

## 3. Tests and Surveys

As previously mentioned, an initial comparison between the two systems, the Tromino^®^ and the AlN-MEMS device, was conducted. Specifically, acquisitions with both systems were performed under controlled conditions, where vibrations with imposed amplitude and frequency were generated. Subsequently, acquisitions were carried out directly in specific locations of interest inside the Castello Ursino for the detection of kinetic energy to confirm the efficiency of the self-powered device as a vibration sensor.

### 3.1. Laboratory TEST

#### 3.1.1. The AlN-MEMS

**Figure 2:**
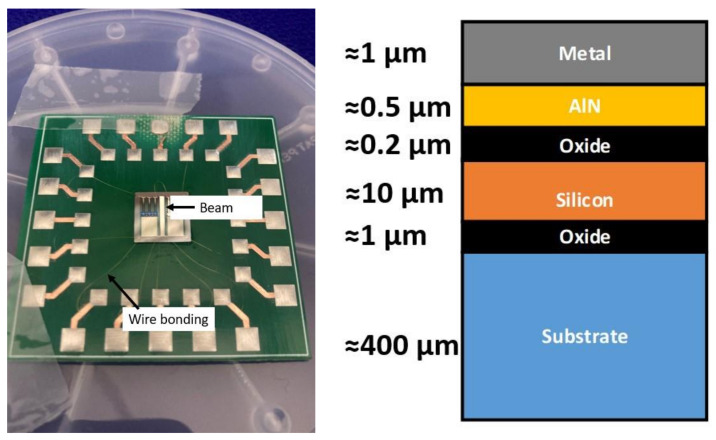
On the left, the AlN-MEMS developed. The picture shows the beam used as a generating transducer through the AlN, and the 1 mil wire bonding used to contact the chip (about 1 cm × 1 cm) to a printed circuit board (PCB) to connect the device. On the right is a detailed image of the foundry process and the layers involved.

Characterization and comparison between the commercial Tromino^®^ instrument and the AlN-MEMS device developed in the same department were conducted at the DIEEI electrical measurements laboratory of the University of Catania. This type of MEMS utilizes aluminum nitride as the piezoelectric material (see Figure 2) and is well regarded for its low cost and self-powered capabilities. The process utilizes a SOI wafer composed of a 10 µm thick doped silicon layer on top, a 400 µm thick substrate, and a 1 µm buried oxide layer. To fabricate suspended structures and perform surface micromachining on the 10 µm silicon layer, a DRIE back-etching technique was employed. An AlN layer was used as the active material for converting mechanical displacement into an electrical signal. Additionally, 1 µm of aluminum and 20 nm of chromium were deposited in order to contact the AlN layer. Figure 2 provides a detailed view of the designed device. This AlN-MEMS device represents a low-cost solution [39] for vibration measurements, being self-powered by the same vibrations it measures; the adoption of the AlN also satisfies the lower power budget of the measurement system and is therefore suitable for long-term monitoring.

The transducer consists of a cantilever beam characterized by a length (l) of about 7.2 mm, a width (w) of 0.8 mm, and a thickness (t) given by the sum of multiple layers, specifically the two metallic layers (Al and Cr), the piezoelectric layer (AlN), and silicon. Its natural angular frequency, considering the AlN young modulus (E_AlN_), which is the maximum of the stacked layers presented in Figure 2, and the mass (M) of the microsystem, can be expressed as follows:

(4) $$\omega_{n}\approx \sqrt{\frac{E_{\mathrm{AlN}}{\;t}^{3}\;w}{4{\;l}^{3}\;M}}$$

#### 3.1.2. Setup

**Figure 3:**
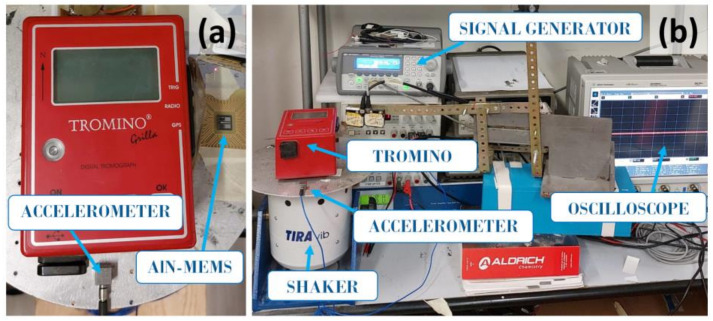
(**a**) Tromino^®^, AlN-MEMS, and an accelerometer positioned on the shaker plate; (**b**) tools employed to characterize the AlN-MEMS and compare it with the Tromino^®^ system and a commercial accelerometer.

An experimental setup was used to compare the signal recorded by the AlN-MEMS and the Tromino^®^ (Figure 3a) by imposing a known vibration level, measuring the output voltage, and comparing the signals with the response of a commercial accelerometer. Figure 3b illustrates the experimental setup used. It includes a TIRA electrodynamic shaker (TV51110) used to excite the transducers with known vibrations following sinusoidal waveforms generated by a signal generator (Agilent 33120A). The commercial feedback accelerometer (PCB333B40-SN51174) is connected to a power supply. Data measurements were collected using an Agilent MSO9064A oscilloscope. During the characterization process, various excitation amplitudes were tested at different frequencies (between 10 Hz and 120 Hz).

**Figure 4:**
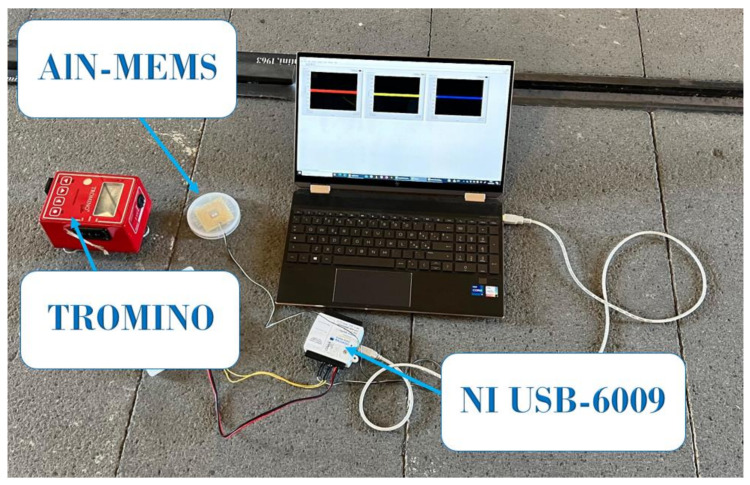
Tromino^®^, AlN-MEMS and the acquisition system positioned on the floor at a chosen point of the Castello Ursino.

For the acquisition of the voltage signals generated by the MEMS during the experimental tests at Castello Ursino, a National Instruments USB-6009 acquisition board was used, connected via a USB cable to a battery-powered laptop (see Figure 4). This setup was chosen to avoid disturbances caused by fluctuations in the power supply current. An automatic data acquisition and saving procedure was developed in LabVIEW, the integrated visual programming development environment established by National Instruments.

### 3.2. Vibration Surveys at Castello Ursino

Vibration measurements were conducted in the Picture Gallery of Castello Ursino, located on the second level (Figure 1b,c), on a wooden mezzanine secured to the castle walls. Two distinct acquisition campaigns were carried out, with a 4-month temporal gap between them, ensuring the alignment of the acquisition points. During the initial survey, only Tromino^®^ devices were used to acquire data from Cu1 to Cu4 (Figure 1c, the acquisition points are indicated by the red dots) for a duration of 75 min at a sampling frequency of 512 Hz. In the subsequent survey, the measurements were repeated twice: first using only the Tromino^®^ device and then simultaneously using both Tromino^®^ and AlN-MEMS devices, for a duration of 30 min, maintaining the same sampling frequency. In Figure 1c, the acquisition points are indicated by blue dots: Cu2.R to Cu4.R denote the data acquired exclusively by the Tromino^®^; Cu1.M to Cu4.M represent the measurements acquired with the Tromino^®^, while Cu1.MEMS to Cu4.MEMS indicate the measurements acquired with the AlN-MEMS during the simultaneous acquisition. The purpose of this repeated investigation over time was to verify any structural changes by comparing the modal analysis results and to evaluate the operation of the Tromino^®^ and AlN-MEMS devices.

Among the acquisition points, three were selected on the lateral sides of the mezzanine, while one was chosen in the central part (Cu4, Cu4.M and Cu4.MEMS in Figure 1c). This decision was based on the observation that the central part typically exhibits a different dynamic behavior compared to the lateral ones and is subject to greater movements.

To reduce potential interferences, the data collection sessions were planned for the afternoon, ensuring minimal visitor activity in the castle museum and no guided tours.

## 4. Data Analysis

### 4.1. Tromino^®^ Data Analysis

#### 4.1.1. Spectral Analysis Results

The Acceleration Peaks consistently occurred around two key frequency values: one at a high frequency and the other at a low frequency (Table 1). The low frequency was approximately 12 Hz with an average acceleration peak of 0.151 mm/s^2^. The high frequency was approximately 100 Hz with an average acceleration peak of 0.083 mm/s^2^. These findings suggest the presence of specific structural resonances at these frequencies, indicating the potential modes of vibration or relevant structural characteristics.

**Figure 5:**
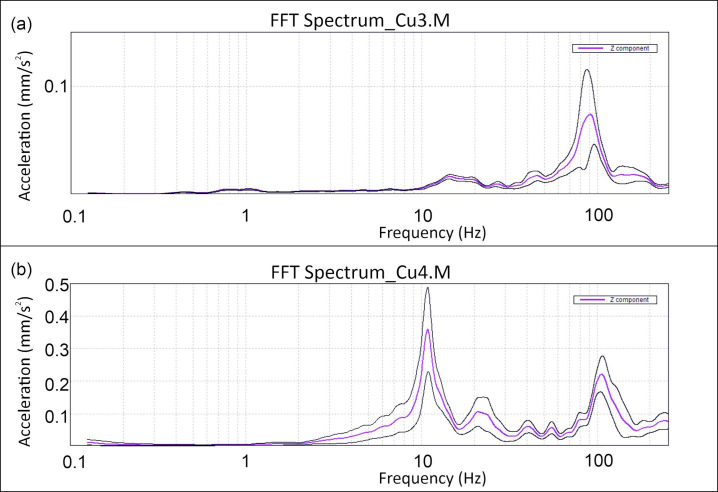
Spectra for the vertical component of the vibration measurement of the (**a**) Cu3.M and (**b**) Cu4.M measurement points: acceleration is expressed on a linear scale, while frequency is presented on a logarithmic scale; black lines represent the standard deviation.

However, the energy levels, denoted by the acceleration peak values at these specific frequencies, exhibited significant variability within the room. This is demonstrated by the fact that in some measurements, such as Cu1.M, Cu3.R, and Cu3.M, the peak at the higher frequency surpasses that at the lower frequency, while in other measurements, the reverse is true (Figure 5).

The maximum acceleration peaks are observed in the central sector of the room at the measurement Cu4.M (Figure 5b), which reaches an acceleration peak of 0.36 mm/s^2^ at a frequency of 11 Hz (Table 1). This evidence demonstrates that higher vibrations occur in the central sector compared to the lateral ones.

#### 4.1.2. PSD Analysis Results

**Figure 6:**
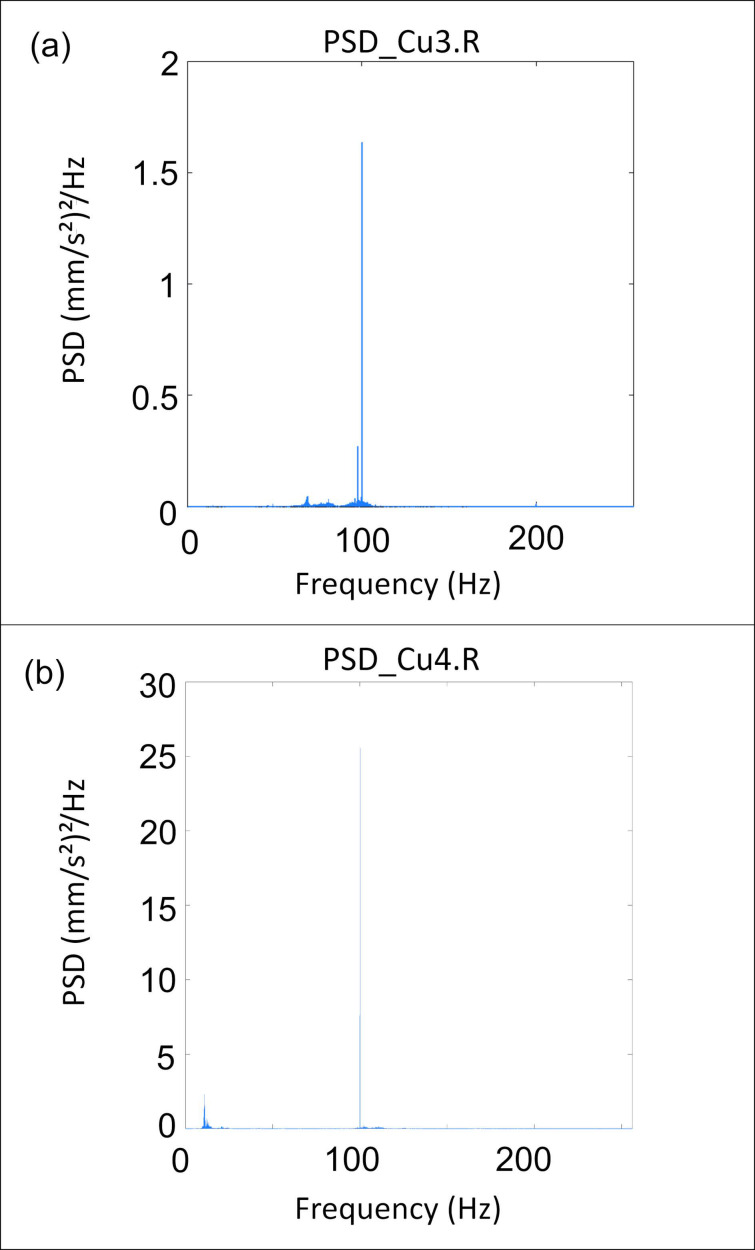
PSD graphs of the (**a**) Cu3.R and (**b**) Cu4.R measurement positions.

The analysis revealed variable PSD values, ranging from very low to very high (Table 2 and Figure 6). The data presented in Table 2 indicate that the PSD peaks are primarily concentrated around two distinct frequency ranges: a lower frequency range around 13 Hz and a higher frequency range around 100 Hz. For instance, the Cu2.R measurement shows a PSD peak of 3.40 (mm/s^2^)^2^/Hz at 13.13 Hz and 5.35 (mm/s^2^)^2^/Hz at 100.04 Hz, indicating significant vibrational energy at both a low and a high frequency. Similarly, the Cu4.R measurement has a notable PSD peak of 25.57 (mm/s^2^)^2^/Hz at 100.05 Hz (Figure 6b), highlighting the predominance of high-frequency vibrations in this particular location. This distribution suggests that the vibrational energy in the environment being monitored is notably significant at these two characteristic frequencies where the structure experiences the most vibrational energy.

The PSD values exhibit variability within the same frequency ranges. For frequencies around 13 Hz, the PSD values range from 3.40 to 5.57 (mm/s^2^)^2^/Hz, and for frequencies around 100 Hz, they range from 0.24 to 25.57 (mm/s^2^)^2^/Hz. This variability highlights the differences in the vibrational energy levels across the measurements. In general, the analysis shows that the average PSD values are higher at frequencies around 100 Hz compared to those around 13 Hz. Specifically, the average PSD value around 13 Hz is 4.485 (mm/s^2^)^2^/Hz, while the average PSD value around 100 Hz is 5.794 (mm/s^2^)^2^/Hz. This indicates that the vibrational energy is generally more pronounced at higher frequencies.

The measurements with the highest PSD values, with Cu4.M recording a peak of 30.47 (mm/s^2^)^2^/Hz at a frequency of 120.13 Hz and Cu4.R recording a peak of 25.57 (mm/s^2^)^2^/Hz at a frequency of 100.05 Hz, indicate that, in the central sector, the structure experiences maximum kinetic energy and that this occurs for a frequency around 100 Hz.

#### 4.1.3. Acceleration Peak Analysis and RMS Analysis Results

The data presented in Table 3 indicate the maximum acceleration peaks and RMS values for various measurements. These metrics are crucial for assessing the vibrational energy levels at different locations within the monitored environment.

**Figure 7:**
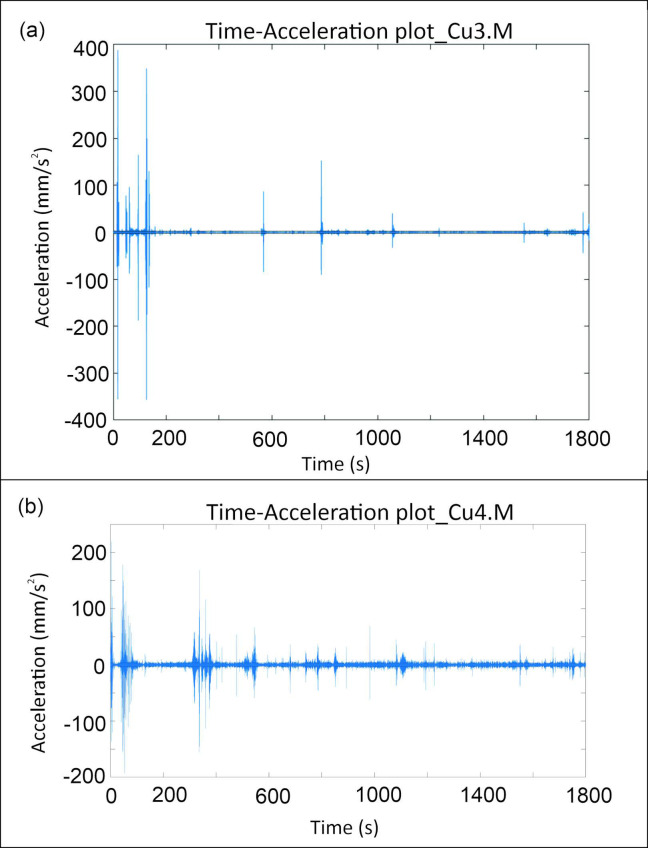
The Time–Acceleration plots for the following measurements: (**a**) Cu3.M; (**b**) Cu4.M.

The measurements for Cu2.M and Cu3.M (Figure 7a) stand out with significantly higher acceleration peaks and RMS values, indicating areas of elevated vibrational energy. Specifically, Cu2.M exhibits a maximum acceleration peak of 365.56 mm/s^2^ and an RMS value of 2.62 mm/s^2^, while Cu3.M shows an even higher peak at 388.07 mm/s^2^ and an RMS value of 2.28 mm/s^2^. Another notable measurement is Cu4.M (Figure 7b), which also displays a high level of vibrational energy, with a maximum acceleration peak of 219.41 mm/s^2^ and the highest RMS value among the measurements at 3.59 mm/s^2^. This indicates that Cu4.M is another critical area with significant vibrational energy.

### 4.2. AlN-MEMS Data Analysis

Field surveys conducted at the art gallery under examination identified three predominant vibration frequencies: 13 Hz, 17 Hz, and 100 Hz. Laboratory tests on the performance of the self-powered device focused mainly on these frequencies, with the mechanical resonant frequency of the MEMS being about 250 Hz. This value is in accordance with Equation (4).

To characterize the device, tests were conducted at frequencies of 13 Hz and 100 Hz. At these frequencies, measurements were taken by applying sinusoidal signals of fixed frequency to the shaker by varying the vibration amplitude of the signal generator used to drive the shaker, with values of 300, 350, 450, and 550 mV for 13 Hz, and 240, 300, 350, and 400 mV for 100 Hz. A maximum corresponding acceleration of about 3.5 m/s^2^ at 14 Hz was measured with the feedback accelerometer.

**Figure 8:**
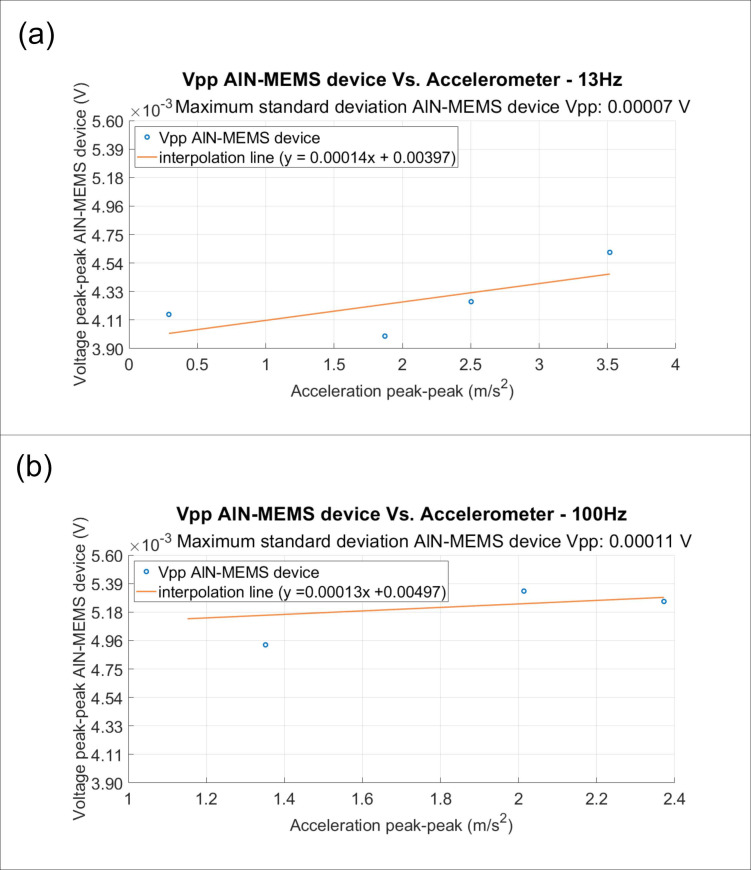
Output voltages from the AlN-MEMS device as a function of the imposed vibration levels: (**a**) at an excitation frequency of 13 Hz and (**b**) at an excitation frequency of 100 Hz. The solid lines, which connect the mean values of the output signal as a function of the acceleration value, have been added to help the eye.

Figure 8 displays the characteristic diagrams at excitation frequencies of 13 Hz (a) and 100 Hz (b). These diagrams present the comparison curves between the peak-to-peak voltage output from the AlN-MEMS device and the imposed peak-to-peak acceleration values. The source of acceleration was the shaker, and the feedback element was the accelerometer, as shown in Figure 3b.

Following the aforementioned characterization, the MEMS was positioned next to the Tromino^®^ at four selected points inside the Castello Ursino (Figure 1b,c). Specifically, the recording of vibrations using both systems was carried out at the points identified by the following labels: Cu1, Cu2, Cu3 and Cu4. In each location, a 30-min recording was made with an acquisition frequency of 1 kHz. The uncertainty estimated was 0.02 mV at 13 Hz and 0.03 mV at 100 Hz.

**Figure 9:**
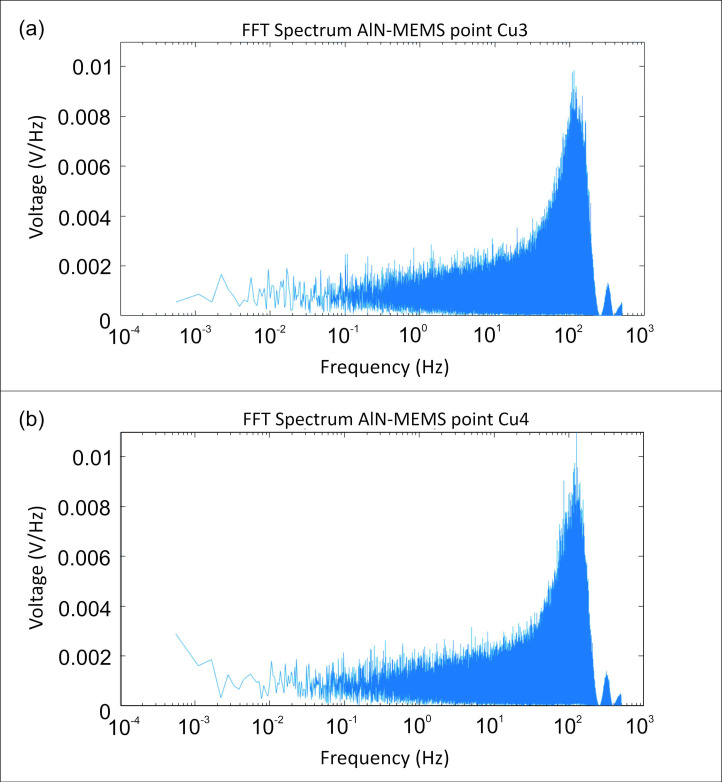
FFT plots for the measurements made by AIN-MEMS at the (**a**) Cu3.M and (**b**) Cu4.M points.

**Figure 10:**
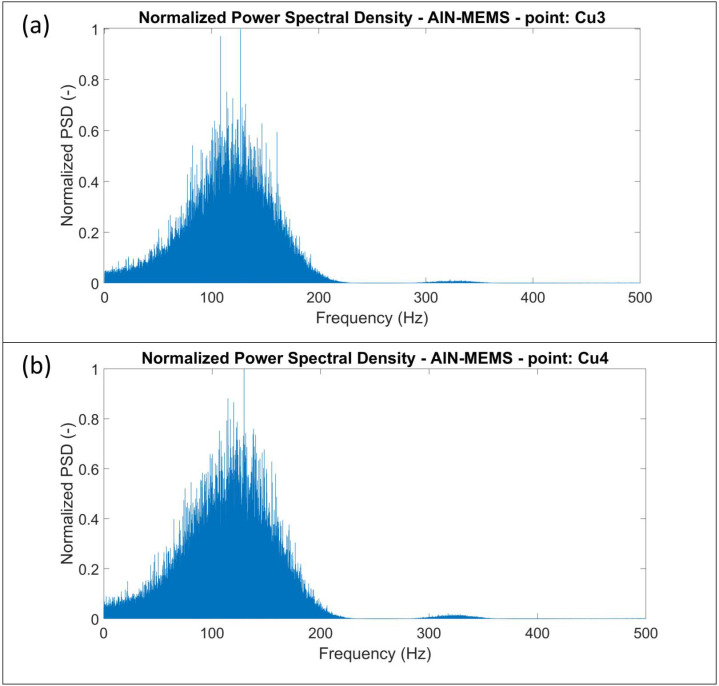
PSD plots for the measurements made by AIN-MEMS at the (**a**) Cu3.M and (**b**) Cu4.M points.

Below are some of the results obtained. Specifically, Figure 9 shows the Spectral Analysis (FFT frequency analysis) of the filtered signal at points Cu3 and Cu4. Figure 10 presents the analysis in terms of the normalized PSD, normalized to its maximum value, at the same points.

## 5. Discussion

The Spectral Analysis and PSD data obtained with the Tromino^®^ reveal that vibrational energy is primarily concentrated around two frequency bands: a lower frequency range (12–13 Hz) and a higher frequency range (100 Hz). Notably, higher energy levels are frequently observed in the higher frequency band across multiple measurements.

The data indicate that the highest energy levels are found in measurements Cu2 and Cu4. Specifically, Cu4 shows significant energy levels, with a PSD peak of 30.47 (mm/s^2^)^2^/Hz around 120.13 Hz and 25.57 (mm/s^2^)^2^/Hz around 100.05 Hz, accompanied by notable acceleration peaks such as 0.36 mm/s^2^ at approximately 10.89 Hz. Similarly, Cu2 displays considerable energy levels, with PSD peaks of 5.57 (mm/s^2^)^2^/Hz around 13.03 Hz and 5.35 (mm/s^2^)^2^/Hz around 100.04 Hz, alongside substantial acceleration peaks of 0.28 mm/s^2^ at approximately 12.52 Hz. These data highlight that Cu2 and Cu4 are critical points of vibrational energy, suggesting that these locations are optimal for monitoring and energy-harvesting applications due to their high kinetic energy levels.

The analysis of the vibrational acceleration over time indicates that the highest vibrational energy levels are observed at location Cu4, followed by Cu2 and Cu3. The data show that Cu4 exhibits the most significant vibrational energy, with a combined maximum acceleration peak of 219.41 mm/s^2^ and the highest RMS value of 3.59 mm/s^2^. This suggests that the measurement site at Cu4 is subjected to substantial dynamic activity, making it a crucial point for monitoring. Following Cu4, the Cu2 location also shows high vibrational energy levels, with a maximum acceleration peak of 365.56 mm/s^2^ and an RMS value of 2.62 mm/s^2^. The Cu3 location similarly indicates significant vibrational activity, with a maximum acceleration peak of 388.07 mm/s^2^ and an RMS value of 2.28 mm/s^2^. These measurements highlight the importance of these locations for strategic sensor placement to monitor and employ ambient energy effectively. The sustained high-energy vibrations at these points underscore their significance for the deployment of AlN-MEMS sensors, ensuring optimal vibration monitoring and energy-harvesting applications. The results obtained from the MEMS show a response comparable to that of the commercial device, particularly around 100 Hz. These findings demonstrate the capability of the MEMS device to measure vibrations in the museum environment in a self-generating manner, thus eliminating the need for batteries or conditioning circuits. The obtained results are consistent with those from the Tromino^®^ device, confirming the MEMS’ sensing properties.

Additionally, the MEMS device can utilize the transduction properties of AlN to harvest energy from environmental vibrations, achieving a power density of approximately 0.28 mW/cm^3^ at the resonant frequency. It is worth noting that the MEMS under study produces significant output voltage values compared to other generating materials, such as BaTiO_3_ and ZnO nanowires [40].

It is worth noting that the device used does not require conditioning circuits, as it already has an output in volts. Therefore, it is suitable for direct processing in the case of sensing, or appropriately managed in the case of harvesting using solutions already known in the literature for generating systems [41,42].

## 6. Conclusions

The primary objective of this study was to conduct vibration analysis at the Castello Ursino Picture Gallery (Sicily, Italy) and to explore the potential of using self-powered AlN-MEMS sensors for capturing environmental vibrations within the gallery.

The spectral analysis and PSD data consistently indicated that the vibrational energy is concentrated around two frequency ranges: approximately 12–13 Hz and 100 Hz. The measurements highlighted specific locations, particularly Cu4, as exhibiting the highest vibrational energy intensity levels. This suggests that these points are optimal for the placement of MEMS sensors to ensure efficient vibration monitoring and energy harvesting.

Furthermore, the comparison between the AlN-MEMS sensors and the commercial Tromino^®^ device demonstrated that the AlN-MEMS sensors provide a comparable performance in measuring vibrations, especially around 100 Hz. The findings confirm that AlN-MEMS sensors are capable of effectively monitoring vibrations and converting kinetic energy into electrical power, thereby eliminating the need for external power sources.

In summary, the results emphasize the importance of strategically placing MEMS sensors in areas with significant vibrational energy. The successful application of AlN-MEMSs in this study underscores their potential for enhancing the monitoring and preservation of historical structures by providing a reliable, self-sustaining solution for capturing and analyzing environmental vibrations.

Work is in progress to improve the performance of the MEMS, including its bandwidth. To this end, we are exploring new designs to widen the bandwidth, including nonlinear modes and MEMS configurations with a tunable frequency. Ongoing work also aims to validate the MEMS device in other areas of interest, ensuring its reliability and performance in broader applications related to vibration analysis.

## Figures and Tables

**Table 1:** Frequency values from spectral analysis and corresponding acceleration peaks.

Measurement	Frequency (Hz) from Spectral Analysis	Acceleration Peak (mm/s^2^) for Frequency (Hz) from Spectral Analysis
Cu1.M	11.20	0.016
	15.22	0.015
	106.38	0.016
	136.72	0.019
Cu2.R	12.87	0.179
	107.87	0.051
Cu2.M	12.52	0.280
	16.78	0.130
	104.90	0.110
Cu3.R	14.29	0.016
	68.56	0.037
	98.53	0.048
Cu3.M	14.19	0.020
	90.62	0.070
Cu4.R	10.97	0.190
	102.74	0.068
Cu4.M	10.89	0.360
	104.91	0.220

**Table 2:** Power Spectral Density peak values and corresponding frequencies.

Measurement	Frequency (Hz)	PSD Peak (mm/s^2^)^2^/Hz
Cu1.M	100.04	0.39
Cu2.R	13.13	3.40
	100.04	5.35
Cu2.M	13.03	5.57
	100.06	2.97
Cu3.R	100.04	1.64
Cu3.M	93.21	0.25
	100.00	0.24
Cu4.R	100.05	25.57
Cu4.M	100.04	9.98
	120.13	30.47

**Table 3:** Peak acceleration values and RMS values.

Measurement	Maximum Acceleration Peak (mm/s^2^)	RMS (mm/s^2^)
Cu1.M	125.40	0.55
Cu2.R	115.51	1.93
Cu2.M	365.56	2.62
Cu3.R	20.40	0.53
Cu3.M	388.07	2.28
Cu4.R	40.66	1.42
Cu4.M	219.41	3.59

## Data Availability

The data are available upon request from the corresponding authors.

## References

[1] Boscato G., Dal Cin A., Russo S., Sciarretta F. (2014). SHM of historic damaged churches. Adv. Mat. Res.

[2] Brownjohn J.M.W. (2006). Structural health monitoring of civil infrastructure. Philos. Trans. R. Soc. A.

[3] (2014). Criteri di Misura e Valutazione Degli Effetti Delle Vibrazioni Sugli Edifici (DIN 4150-3).

[4] (2017). Misura Delle Vibrazioni Negli Edifici e Criteri di Valutazione del Disturbo.

[5] Smyth A.W., Brewick P., Greenbaum R., Chatzis M., Serotta A., Stünkel I. (2016). Vibration mitigation and monitoring: A case study of construction in a museum. J. Am. Inst. Conserv..

[6] Rainer J.H. (1982). Effect of vibrations on historic buildings: An overview. Bull. Assoc. Preserv. Technol..

[7] Rainieri C., Fabbrocino G. (2014). Operational Modal Analysis of Civil Engineering Structures.

[8] Pirrotta A., Russotto S. (2023). A new OMA method to perform structural dynamic identification: Numerical and experimental investigation. Acta Mech..

[9] Ewins D.J. (2009). Modal Testing: Theory, Practice and Application.

[10] Au S.K. (2017). Operational Modal Analysis Modeling, Bayesian Inference, Uncertainty Laws.

[11] Ozbek M., Rixen D.J. (2013). Operational modal analysis of a 2.5 mw wind turbine using optical measurement techniques and strain gauges. Wind. Energy.

[12] Jiang S.F., Qiao Z.H., Li N.L., Luo J.B., Shen S., Wu M.H., Zhang Y. (2020). Structural Health Monitoring System Based on FBG Sensing Technique for Chinese Ancient Timber Buildings. Sensors.

[13] Minardo A., Persichetti G., Testa G., Zeni L., Bernini R. (2012). Long term structural health monitoring by Brillouin fibre-optic sensing: A real case. J. Geophys. Eng..

[14] Nöther N., Wosniok A., Krebber K. (2008). A Distributed fiber optic sensor system for dike monitoring using Brillouin frequency domain analysis. Proceedings Volume 7003, Optical Sensors 2008.

[15] Peeters B., Gajdatsy P., Aarnoutse P., Janssens K., Desmet W. Vibroacoustic operational modal analysis using engine run-up data. Proceedings of the IOMAC 2009-3rd International Operational Modal Analysis Conference.

[16] Rinaldi C., Ciambella J., Gattulli V. (2022). Image-based operational modal analysis and damage detection validated in an instrumented small-scale steel frame structure. Mech. Syst. Signal Process..

[17] Abdullahi S.I., Che Mustapha N.A., Habaebi M.H., Islam M.R. (2019). Accelerometer Based Structural Health Monitoring System on the Go: Developing Monitoring Systems with NI LabVIEW. Int. J. Online Biomed. Eng..

[18] Warsi Z.H., Irshad S.M., Khan F., Shahbaz M.A., Junaid M., Amin S.U. Sensors for Structural Health Monitoring: A Review. Proceedings of the 2019 Second International Conference on Latest Trends in Electrical Engineering and Computing Technologies (INTELLECT).

[19] Ozevin D. (2022). Microelectromechanical systems for assessing and monitoring civil infrastructures. Sensor Technologies for Civil Infrastructures.

[20] Kumar J., Bajpai R. (2012). Application of Mems in Bridge Structures Health Monitoring. Int. J. Eng. Innov. Technol. (IJEIT).

[21] Hossain M.I., Zahid M.S., Chowdhury M.A., Hossain M.M.M., Hossain N. (2023). MEMS-based energy harvesting devices for low-power applications–a review. Results Eng..

[22] Kang J.G., Kim H., Shin S., Kim B.S. (2024). Fluid flow to electricity: Capturing flow-induced vibrations with micro-electromechanical-system-based piezoelectric energy harvester. Micromachines.

[23] Ejeian F., Azadi S., Razmjou A., Orooji Y., Kottapalli A., Ebrahimi Warkiani M., Asadnia M. (2019). Design and Applications of MEMS Flow Sensors: A Review. Sens. Actuators A Phys..

[24] Tuoi T.T.K., Van Toan N., Ono T. (2023). Thermal energy harvester using ambient temperature fluctuations for self-powered wireless IoT sensing systems: A review. Nano Energy.

[25] Hao D., Qi L., Tairab A.M., Ahmed A., Azam A., Luo D., Pan Y., Zhang Z., Yan J. (2022). Solar energy harvesting technologies for PV self-powered applications: A comprehensive review. Renew. Energ..

[26] Muscalu G., Firtat B., Anghelescu A., Moldovan C., Dinulescu S., Brasoveanu C., Ekwinska M., Szmigiel D., Zaborowski M., Zajac J. (2024). Piezoelectric MEMS Energy Harvester for Low-Power Applications. Electronics.

[27] (2020). Moho. TROMINO Users Manual, p. 144. https://moho.world/en/.

[28] Grassi S., Barbano M.S., Pirrotta C., Morreale G., Imposa S. (2022). Seismic Soil–Structure Interaction of Three Historical Buildings of the University of Catania (Sicily, Italy). Heritage.

[29] Grassi S., Patti G., Tiralongo P., Imposa S., Aprile D. (2021). Applied geophysics to support the cultural heritage safeguard: A quick and non-invasive method to evaluate the dynamic response of a great historical interest building. J. Appl. Geophys..

[30] Imposa G., Grassi S., Barontini A., Morreale G., Russo S., Lourenço P.B., Imposa S. (2023). Extended Tromograph Surveys for a Full Experimental Characterisation of the San Giorgio Cathedral in Ragusa (Italy). Sensors.

[31] Imposa S., Cuomo M., Contrafatto L., Mineo S., Grassi S., Li Rosi D., Barbano M.S., Morreale G., Galasso M., Pappalardo G. (2023). Engineering Geological and Geophysical Studies Supporting Finite Element Analysis of Historical Buildings after Dynamic Identification. Geosciences.

[32] Paolucci E., Albarello D., D’Amico S., Lunedei E., Martelli L., Mucciarelli M., Pileggi D. (2015). A large scale ambient vibration survey in the area damaged by May–June 2012 seismic sequence in Emilia Romagna, Italy. B. Earthq. Eng..

[33] Gueli A.M., Imposa S., Mancuso B., Pinto V., Pirrotta C., Politi G., Salerno G.A., Trigona C. (2024). Castello Ursino Museum’s Structural Monitoring Enhanced by Self-Energized Solutions. Proceedings of the 21st International Multi-Conference on Systems, Signals & Devices (SSD).

[34] Jwo D.J., Chang W.Y., Wu I.H. (2021). Windowing techniques, the Welch Method for improvement of power spectrum estimation. Comput. Mater. Contin..

[35] Welch P.D. (1967). The use of Fast Fourier Transform for the estimation of power spectra: A method based on time averaging over short, modified periodograms. IEEE Trans. Acoust. Speech.

[36] Nussbaumer H.J. (1982). The Fast Fourier Transform. Fast Fourier Transform and Convolution Algorithms.

[37] Monson H.H. (1996). Statistical Digital Signal Processing and Modeling.

[38] Stoica P., Randolph M. (2005). Spectral Analysis of Signals.

[39] Trigona C., Sinatra V., Crea G., Andò B., Baglio S. (2019). Characterization of a PiezoMUMPs microsensor for contactless measurements of DC Electrical current. IEEE Trans. Instrum. Meas..

[40] Alrashdan M.H., Hamzah A.A., Majlis B.Y. (2018). Power density optimization for mems piezoelectric micro power generator below 100 Hz applications. Microsyst. Technol..

[41] Chéour R., Jmal M.W., Khriji S., El Houssaini D., Trigona C., Abid M., Kanoun O. (2021). Towards hybrid energy-efficient power management in wireless sensor networks. Sensors.

[42] Giusa F., Maiorca F., Noto A., Trigona C., Andò B., Baglio S. (2014). A diode-less mechanical voltage multiplier: A novel transducer for vibration energy harvesting. Sens. Actuators A Phys..

